# Phytochemical Profile and Antidiabetic, Antioxidant, and Anti-Inflammatory Activities of *Gypsophila paniculata* Ethanol Extract in Rat Streptozotocin-Induced Diabetes Mellitus

**DOI:** 10.3390/antiox13091029

**Published:** 2024-08-24

**Authors:** Lia-Oxana Usatiuc, Marcel Pârvu, Raluca Maria Pop, Ana Uifălean, Dan Vălean, Csilla-Eniko Szabo, Mădălina Țicolea, Florinela Adriana Cătoi, Floricuța Ranga, Alina Elena Pârvu

**Affiliations:** 1 Pathophysiology, Department 1—Morphofunctional Sciences, Faculty of Medicine, “Iuliu Hațieganu” University of Medicine and Pharmacy, 400012 Cluj-Napoca, Romania; lia.usatiuc@umfcluj.ro (L.-O.U.); ana.uifalean@umfcluj.ro (A.U.); madalinaticolea@umfcluj.ro (M.Ț.); adriana.catoi@umfcluj.ro (F.A.C.); parvualinaelena@umfcluj.ro (A.E.P.); 2 Department of Taxonomy, Faculty of Biology and Geology, “Babes-Bolyai” University, 400012 Cluj-Napoca, Romania; marcel.parvu@ubbcluj.ro; 3 Pharmacology, Toxicology and Clinical Pharmacology, Department 1—Morphofunctional Sciences, Faculty of Medicine, “Iuliu Hațieganu” University of Medicine and Pharmacy, 400012 Cluj-Napoca, Romania; 4 Surgery Department, “Iuliu Haţieganu” University of Medicine and Pharmacy, 400012 Cluj-Napoca, Romania; valean.d92@gmail.com; 5 Pediatric Clinic 1, Department of Mother and Child, “Iuliu Hatieganu” University of Medicine and Pharmacy, 400012 Cluj-Napoca, Romania; csilla.szabo@umfcluj.ro; 6 Food Science and Technology, Department of Food Science, University of Agricultural Science and Veterinary Medicine Cluj-Napoca, Calea Mănăștur, No 3-5, 400372 Cluj-Napoca, Romania; florica.ranga@usamvcluj.ro

**Keywords:** *Gypsophila paniculata*, diabetes mellitus, oxidative stress, inflammation

## Abstract

The present study aimed to investigate the effects of the *Gypsophila paniculata* ethanol extract (GPEE) on oxidative stress, inflammation, and metabolic markers in a rat model of streptozotocin-induced diabetes mellitus (DM). Phytochemical analysis using high-performance liquid chromatography coupled with mass spectrometry was performed to measure the total phenolic and flavonoid contents. In vitro antioxidant activity was evaluated through DPPH, FRAP, H_2_O_2_, and NO scavenging tests, and the in vivo effects of the GPEE were assessed in streptozotocin-induced DM rats. Treatments with the GPEE, metformin, and Trolox were administrated by gavage for 10 days. On day 11, blood was collected, and serum oxidative stress (total oxidative status, oxidative stress index, malondialdehyde, advanced oxidation protein products, 8-hydroxydeoxyguanosine, nitric oxide, 3-nitrotyrosine, advanced glycation end-products, total antioxidant reactivity, total thiols), inflammatory (IL-1β, NF-κB, IL-18, and gasdermin D), metabolic (fasting glucose, total cholesterol, triglycerides, and triglyceride–glucose index), and liver injury (AST, ALT, and AST:ALT ratio) markers were measured. The GPEE was found to have a significant polyphenols content and a moderate in vitro antioxidant effect. In vivo, the GPEE lowered oxidants and increased antioxidants, decreased inflammatory markers and blood glucose, and improved lipid profiles and transaminases in a dose-dependent manner, with higher doses having a better effect, being comparable to those of metformin and Trolox.

## 1. Introduction

Diabetes mellitus is a complex metabolic disease with a multifactorial etiology, significantly impacting global morbidity and mortality, and imposing substantial economic and social burdens. In DM, long-term hyperglycemia results from anomalies in insulin secretion, insulin action, or both, manifesting as chronic and heterogeneous carbohydrate, lipid, and protein metabolic dysfunctions.

Hyperglycemia-induced oxidative stress (OS), along with enhanced reactive oxygen species (ROS) generation and decreased antioxidant levels, plays a crucial role in the pathogenesis of DM and its associated complications [1,2].

Another mechanism recognized as playing a key role in the pathogenesis and related complications of DM is systemic low-grade chronic inflammation with inflammasome activation. ROS and multiple DM-related metabolic changes may trigger an inflammatory response and inflammasome activation. Furthermore, at the same time, inflammation may increase OS, forming a vicious circle between inflammation and OS [3]. As a consequence of the involvement of inflammation and OS in the pathogenesis of DM, these mechanisms have become therapeutic targets for DM and its associated complications.

At present, there is no cure for DM, and synthetic oral hypoglycemic drugs, together with insulin, cannot completely succeed in preventing the occurrence of complications associated with diabetes, especially considering their adverse effects [4]. Cases where available drugs were not effective have also been reported [5]. Therefore, many studies have searched for evidence on the efficiency of phytotherapies in preventing and controlling DM, as an alternative treatment or as an adjunctive therapy to drugs used for the treatment of DM [6].

To date, a series of phytoconstituents with hypoglycemic properties have been identified. Their mechanisms of action, demonstrated through in vitro enzymatic studies, were similar to those of certain oral antidiabetics, such as alpha-glucosidase or alpha-amylase inhibitors, incretin mimetics, dipeptyl-dipeptidase IV inhibitors, or GLP-1 agonists [7].

*Gypsophila paniculata*, also known as baby’s breath, common gypsophila, or panicled baby’s breath, is an herbaceous plant of the Caryophyllaceae family native to central and eastern Europe. In traditional medicine, *G. paniculata* has been used to manage a wide range of diseases, including respiratory conditions (due to its potential expectorant and bronchodilator properties) and gastrointestinal diseases (due to its gastroprotective and purgative effects). Previous in vitro studies have reported the antimicrobial, antioxidant, and antitumoral activities of *G. paniculata* [8,9]. The aerial parts of Gypsophila species (e.g., *G. oldhamiana*, *G. glomerata*, *G. trichotoma*, and *G. perfoliate*) have been previously reported as novel sources of bioactive agents with antidiabetic properties [10,11]. The main bioactive components are represented by triterpene saponins [8]. Among these, gypsogenin has been shown to inhibit glycogen phosphorylase (GP), a key enzyme involved in glycogen breakdown [12]. Other natural triterpenoids have been shown to exhibit a wide spectrum of pharmacological activities against DM, presenting multi-target pharmaceutical effects such as stimulating insulin secretion while reversing insulin resistance at the same time, as well as antioxidant and anti-inflammatory activities [13]. While there is limited information available on the specific antidiabetic and anti-inflammatory activities, as well as the in vivo antioxidant activity of *G. paniculata*, given the promising potential of the bioactive compounds previously isolated from this plant, a better characterization of the in vitro and in vivo effects of *G. paniculata* is needed.

The aim of our study was to analyze the effects of the *G. paniculata* ethanol extract (GPEE) on OS, inflammation, and associated glucose and lipid metabolism dysfunctions in a streptozotocin-induced rat DM model. At the same time, a phytochemical analysis of the GPEE was performed, and the antioxidant activity and liver toxicity of the GPEE were tested in vitro.

## 2. Materials and Methods

### 2.1. Chemicals 

Folin–Ciocâlteu reagent, sodium carbonate, sodium acetate, aluminum chloride, methanol, 2,2-diphenyl-1-picrylhydrazyl (DPPH), Griess–Ilosvay nitrite reagent, sodium nitroprusside, phosphate-buffered saline, N-(1-naphthyl) ethylenediamine dihydrochloric acid (NEDD), sulphanilic acid, hydrogen peroxide (H_2_O_2_), 2,4,6-tri(2-pyridyl)-1,3,5-triazine (TPTZ), acetate buffer, ferric chloride, xylenol orange, ortho dianisidine dihydrochloride (3-3′-dimethoxybenzidine), thiobarbituric acid, ethylenediaminetetraacetic acid, sodium dodecyl sulfate, butylated hydroxytoluene, thiobarbituric acid, 1,1,3,3-tetrahydroxypropane, vanadium III chloride (VCl3), 5,5′-di-thio-bis 2-nitrobenzoic acid (DTNB), and Trolox (6-hydroxy-2,5,7,8-tetramethylchroman-2-carboxylic acid) were purchased from Merck (Darmstadt, Germany) and Sigma–Aldrich (Munich, Germany). Acetonitrile of HPLC purity was purchased from Merck (Darmstadt, Germany), and ultrapure water was purified using the Direct-Q UV system from Millipore (St. Louis, MI, USA). Standard chlorogenic acid (>98% HPLC), ellagic acid (>99% HPLC), rutin, catechin, and cyanidin (>99% HPLC) were purchased from Sigma (St. Louis, MO, USA). NF-κB p65, IL-1β, 3NT, and 8OH-dG ELISA kits were purchased from Elabscience Innovation Bionovation Inc. (Houston, TX, USA). NLRP3 inflammasome biomarkers were determined using ELISA kits from ABclonal Technology (Woburn, MA, USA) for IL-18, and from MyBioSource (San Diego, CA, USA) for Gasmderin D. AST, ALT, TC, TG, and blood glucose assays kits were purchased from Spinreact S.A./S.A.U. (Girona, Spain).

### 2.2. Plant Extract Preparation

The plant material of *G. paniculata* Xlence variety was obtained from OZ Planten B.V. (De Kwakel, The Netherlands) in March 2022. Plants were taxonomically identified, and voucher specimens (number 44.12.5.1/04.03.2022) were deposited at the Faculty of Pharmacy’s Department of Pharmaceutical Botany of “Iuliu Hațieganu” University of Medicine and Pharmacy Cluj-Napoca, Romania. Extraction of *G. paniculata* was performed in the Mycology Laboratory of “Babes-Bolyai” University, Cluj-Napoca, Romania, using a modified Squibb repercolation method with 70% ethanol (Merck, Bucuresti, Romania), producing a 100 mg/1 mL (*w*/*v*) extract.

### 2.3. Total Phenolic Content Determination

The total phenolic content (TPC) of the GPEE was spectrophotometrically determined using the Folin–Ciocálteu method with some modifications. A quantity of 2 mL of ethanol extract was diluted 25 times, then mixed with Folin–Ciocâlteu reagent (1 mL) and distilled water (10.0 mL), and further diluted to 25 mL with a 290 g/L solution of sodium carbonate. The samples were incubated in darkness for 30 min, following which the absorbance was measured at 760 nm using a JASCO V-530 (Jasco International Co., Ltd., Tokyo, Japan) UV–vis spectrophotometer. TPC values are expressed as gallic acid equivalents (GAE) (R^2^ = 0.999), mg GAE/g d.w. herbal material [14]. All of the TPC determinations were performed in triplicate.

### 2.4. Total Flavonoid Content Determination

The total flavonoid content (TFC) of the GPEE was determined using a previously described method [15]. Briefly, 5 mL of the extract was mixed with sodium acetate (5.0 mL, 100 g/L), aluminum chloride (3.0 mL, 25 g/L), and made up to 25 mL in a calibrated flask with methanol. The absorbance was measured at 430 nm. The TFC was expressed as quercetin equivalents (R^2^ = 0.999), mg QE/g d.w. herbal material. The assay was performed in triplicate.

### 2.5. HPLC-DAD-ESI MS Analysis

An Agilent 1200 HPLC system equipped with a quaternary pump, solvent degasser, autosampler, and UV–vis detector with photodiode (DAD) coupled with a single quadrupole mass detector (MS; Agilent model 6110; Agilent Technologies, Santa Clara, CA, USA) was used. The separation of the compounds was carried out using a Kinetex XB C18 column, measuring 4.6 × 150 mm, with 5 μm particles (Phenomenex, Torrance, CA, USA). The mobile phases were (A) water + 0.1% acetic acid and (B) acetonitrile + 0.1% acetic acid, for 30 min, at a temperature of 250 °C, with a flow rate of 0.5 mL/min, in the following gradient (expressed in % B): 0 min, 5% B; 0–2 min, 5% B; 2–18 min, 5–40% B; 18–20 min, 40–90% B; 20–24 min, 90% B; 24–25 min, 90–5% B; 25–30 min, 5% B. The spectral values were recorded in the 200–600 nm range for all peaks. The chromatograms were recorded at wavelengths of λ = 280 and 340 nm.

For MS, full scan ESI positive ionization mode was used with the following working conditions: capillary voltage 3000 V, temperature 3500 °C, nitrogen flow 7 L/min, and *m*/*z* 120–1200. Data acquisition and interpretation of results were performed using Agilent ChemStation software (Rev B.04.02 SP1; Agilent Technologies Inc., Palo Alto, CA, USA). For the quantification of phenolic compounds, calibration curves were obtained by injecting 5 different concentrations of standard substances dissolved in methanol: 1. ellagic acid (R^2^ = 0.9978), LOD = 0.35 μg/mL, LOQ = 1.05 μg/mL; 2. gallic acid (R^2^ = 0.9978), LOD = 0.18 μg/mL, LOQ = 0.72 μg/mL; 3. chlorogenic acid (R^2^ = 0.9937), LOD = 0.41 μg/mL, LOQ = 1.64 μg/mL; and 4. rutin (R^2^ = 0.9981), LOD = 0.21 μg/mL, LOQ = 0.84 μg/mL. The equations of the curves were used for quantitative calculation for each phenolic compound.

### 2.6. Evaluation of In Vitro Antioxidant Activity

The antioxidant activity was assessed through four in vitro methods: 2,2-diphenyl-picryl-hydrazil (DPPH), nitric oxide (NO) scavenging activity, H_2_O_2_ scavenging activity, and ferric-reducing antioxidant power (FRAP) assays [15].

First, the effect of the GPEE against the DPPH radical was tested [16]. Two milliliters of sample solution were added to 2 mL of a 0.1 mg/mL DPPH methanol solution. After 30 min of incubation at room temperature in the dark, the absorbance was read at 517 nm. The DPPH radical scavenging activity is expressed as IC50 (μg/mL). The antioxidant activity is expressed as a percentage using the formula: (AA%) = [(OD control − OD sample)/OD control] × 100, where OD represents the optical density. AA% was subsequently converted to Trolox equivalents (TE) using the Trolox (TX) calibration curve of standard solutions (0.5–5 μg/mL). An IC50 of <50 μg TE/mL indicates a very good antioxidant potential, an IC50 of 50–100 μg TE/mL indicates a good antioxidant potential, an IC50 of 100–200 μg TE/mL indicates a weak antioxidant potential, and an IC50 of >200 μg TE/mL means no antioxidant potential [17].

The NO scavenging activity was measured using the Griess reagent and 0.1% *w/v* naphthylethylene–diamine–dihydrochloride. A total of 0.5 mL of the GPEE was added to a mixture containing 2 mL of SNP and 0.5 mL of phosphate-buffered saline (PBS) at pH 7.4. After incubation for 2.5 h at 25 °C, 0.5 mL of the reaction mixture was added to 1 mL sulphanilic acid (0.33% in 20% glacial acetic acid) followed by the addition of naphthylethylene–diamine–dihydrochloride (1 mL of 0.1% *w*/*v*) after 5 min. The resulting solution was vortexed and incubated for a further 30 min. The absorbance of the chromophores was read at 546 nm and the percentage inhibition was calculated using the following formula: % inhibition = (A blank − A sample/A blank) × 100. The results are expressed as IC50 in mgTE/mL.

The H_2_O_2_ scavenging activity was determined by adding the GPEE to distilled water, which was mixed with a H_2_O_2_ solution. After 10 min, absorbance was assessed at 230 nm against a blank solution of phosphate buffer. The percentage of H_2_O_2_ scavenging was calculated using the following formula: % scavenged H_2_O_2_ = (A control − A sample/A control) × 100. The results are expressed as IC50 in mgTE/mL plant extract.

The FRAP method is a colorimetric test based on the reduction of the Fe^3+^ ion to the ferrous iron (Fe^2+^) with 2,4,6-tri(2-pyridyl)-1,3,5-triazine (TPTZ). FRAP reagent was obtained by mixing acetate buffer (0.3 M, pH 3.6), TPTZ (10 mM) in 40 mM HCl, and ferric chloride (20 mM) in a ratio of 10:1:1 (*v*/*v*/*v*). The absorbance was read at 593 nm after 30 min incubation at room temperature, and the results are expressed as mg TE/mL [17].

### 2.7. Pharmacological Studies

#### 2.7.1. Experimental Design

The experiments were performed on adult male Winstar albino rats weighing 200–250 g. The animals were bred in the “Iuliu Hațieganu” University of Medicine and Pharmacy Animal Facility and were kept under controlled conditions (12 h night/day cycle, temperatures of 22–24 °C, and humidity of 55–60%), with free access to a standard pellet-based diet (Cantacuzino Institute, Bucharest, Romania) and water ad libitum.

The animals were divided into 10 groups, each group containing 10 animals: negative control group (CONTROL), a positive control group with diabetes mellitus (DM) induced with streptozotocin (STZ), and several treatment groups: one group with DM receiving Trolox (50 mg/kg b.w.; SZT + TX), one group with DM receiving metformin (100 mg/100 g b.w.; SZT + M), and three groups with DM receiving the GPEE at different concentrations (100%, 50%, 25%; GPEE100%, GPEE50%, GPEE25%, respectively). Over 10 days, starting with the first day, the CONTROL and SZT groups received tap water by gavage (1 mL/d), while the three GPEE groups received the respective dilutions of the GPEE. From day two, daily fasting glycemia was measured using an Accu Check Active compact glucometer, and the rats received 2–3 IU of Insulin Aspart subcutaneously. On day 11, under general anesthesia induced by a mixture of ketamine (70 mg/kg b.w.) and xylazine (10 mg/kg b.w.), blood samples were collected by retro-orbital puncture and the serum was separated and stored at −80 °C until analysis. All animals were sacrificed by means of cervical dislocation upon completion of the study. 

The experimental design was approved by the Institutional Animal Ethical Committee (IAEC) of the “Iuliu Hațieganu” University of Medicine and Pharmacy Cluj-Napoca and by the National Sanitary Veterinary and Food Safety Agency (nr. 302/04.04.2022).

#### 2.7.2. Evaluation of Serum Oxidative Stress Markers

The total oxidative status (TOS) was determined as a global index of serum oxidants, which was measured using a colorimetric assay based on the oxidation of ferrous ion (Fe^2+^) to ferric ion (Fe^3+^) in the presence of oxidant species in an acidic medium, where the measurement of the ferric ion was conducted through reaction with xylenol orange [18]. The method was applied to an automated analyzer, which was calibrated with hydrogen peroxide. The results are expressed as μM H_2_O_2_ Equiv./L.

The total antioxidant capacity (TAC) was determined as a global index of antioxidant defense. It was measured using a colorimetric method based on the neutralization of dianisidyl radicals resulting from the oxidative process of ortho-dianisidyl [19]. Therefore, a standard solution of Fe^2+^-o-dianisidyl underwent the Fenton reaction with a standard solution of H_2_O_2_, forming hydroxyl ·OH radicals. These radicals, in the presence of an acid, oxidized o-dianisidine to dianisidyl radicals. The antioxidant agents in the sample inhibited the oxidation reactions and the appearance of coloration. At the end of the reactions, the color intensity was determined spectrophotometrically. This assay was calibrated using TX, and the results are expressed as mM TE/L.

The oxidative stress index (OSI), a useful indicator of the influence of the tested compounds on the oxidant–antioxidant balance, was calculated using the following formula: OSI (Arbitrary Unit) = TOS (mM H_2_O_2_Equiv/L)/TAR (mM TE/L) [20].

Malondialdehyde (MDA) was measured using thiobarbituric acid, and its serum concentrations are expressed as nM/mL of serum. Briefly, the method consisted of reacting 150 μL of serum with 125 μL of 10% trichloroacetic acid (TCA), 125 μL of 5 mM ethylenediaminetetraacetic acid (EDTA), 125 μL of 8% sodium dodecylsulfate, and 10 μL of 0.5 butylated hydroxytoluene. After vigorous mixing for 30 s, the mixture was incubated for 10 min at room temperature. Then, 500 μL of 0.6% thiobarbituric acid was added and the mixture was heated at 95 °C for 30 min. After cooling to room temperature, the mixture was centrifuged at 10,000 rpm for 10 min. The absorbance of the supernatant was measured at 532 nm. The standard curve was generated with 1,1,3,3-tetrahydroxypropane as the standard (0.3–10 nM/mL) [21].

The serum concentration of nitric oxide (NO) was assessed using its stable end products, namely, nitrites and nitrates. The Griess reaction, preceded by the reduction of nitrates to nitrites (NOx) with Vanadium III, was used as an indirect detection method. Serum NOx concentrations are expressed as μM nitrites/L [22].

Serum 3-nitrotyrosine (3NT), a marker of peroxynitrite formation, and 8-hydroxydeoxyguanosine (8-OHdG), a biomarker of oxidative DNA damage, were measured through enzyme-linked immunosorbent assay (ELISA) methods using commercial kits, and the results are expressed in ng/mL.

Advanced oxidation protein products (AOPPs) were measured using the method developed by Witko-Sarsat et al. [23]. Serum samples and chloramine T as a blank were diluted to 10% in PBS, and potassium iodide and glacial acetic acid were added. The absorbance of the samples was read at 340 nm, and the AOPP concentration is expressed in µM chloramine-T Equiv./L.

Advanced glycation end-products (AGEs) were measured using the ELISA method with a commercial kit, and the results are expressed in U/mL.

The concentration of total thiols (SH) was measured using Ellman’s reagent (5,5′-di-thio-bis (2-nitrobenzoic acid)), followed by spectrophotometric detection of the obtained supernatant at 412 nm. The results are expressed in terms of reduced glutathione (GSH), mM GSH/mL [24].

#### 2.7.3. Evaluation of Serum Inflammatory Markers

Inflammation was assessed through measuring NLRP3 inflammasome activation serum biomarkers, including nuclear factor kappa B-p65 (NF-κB p65), IL-1β, IL-18, and gasdermin D, with ELISA kits according to the manufacturer’s instructions. NF-κB p65, IL-1β, and IL-18 results are expressed in pg/mL, and gasdermin D in ng/mL.

#### 2.7.4. Evaluation of Glucose, Lipid Profile, Triglyceride–Glucose Index, and Anthropometric Markers

Blood glucose levels were measured at 48 h after DM induction in order to confirm hyperglycemia induced by STZ administration. We used 1 mL blood samples collected from the dorsal tail vein and determined the glucose levels using enzymatic strips and an ACCU-Check Active compact glucometer (Roche, Basel, Switzerland). On day 11, venous blood glucose levels from the samples that were collected via retro-orbital puncture were measured using commercial kits, and the results are expressed as mg/dL.

The serum levels of total serum cholesterol (TC) and triglycerides (TG) were assessed spectrophotometrically using commercial kits according to the manufacturer’s instructions, and the results are expressed as mg/dL.

The triglyceride–glucose index (TyG), a surrogate index used to identify insulin resistance in apparently healthy subjects, was calculated at the end of the study according to the formula ln [fasting triglycerides (mg/dL) × fasting glucose (mg/dL)/2]. The TyG index is expressed on a logarithmic scale [25].

The body weight (BW) of the animals was also measured (g) on the first and last days of the experiment, and the difference between them was calculated.

#### 2.7.5. Evaluation of Liver Injury Markers

Hepatocyte injury was assessed through measuring serum transaminases, aspartate aminotransferase (AST), and alanine aminotransferase (ALT) using commercial kits. Results are expressed as U/L. The AST/ALT ratio was also calculated.

### 2.8. Statistical Analysis

The results for normally distributed data are displayed as mean ± standard deviation (SD). Differences between the groups were estimated using a one-way analysis of variance (ANOVA) and post hoc Bonferroni–Holm tests. To evaluate the correlations between the candidate biomarkers, the Pearson test was conducted. A *p* value of <0.05 was regarded as significant. Multivariate analysis of the parameters was performed using Principal Component Analysis (PCA). SPSS v26.0 for Windows (SPSS Inc., Chicago, IL, USA) and Statistica 12 (TIBCO Software, Palo Alto, CA, USA) were used for data management and statistical analysis.

## 3. Results

### 3.1. Phytochemical Analysis

**Figure 1:**
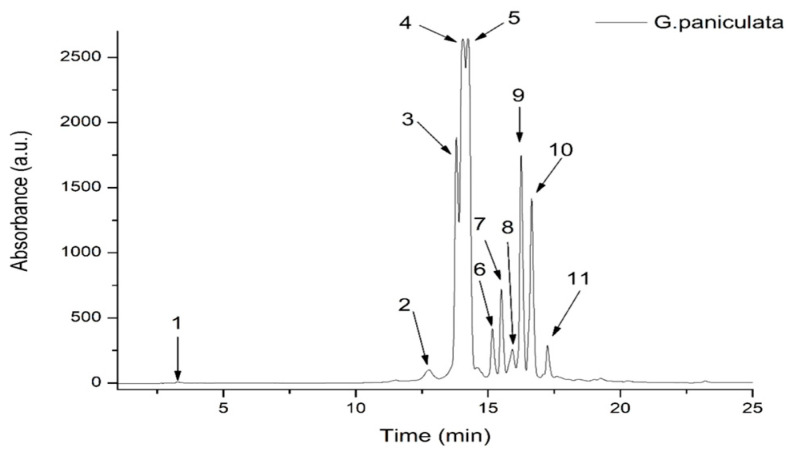
HPLC chromatogram of phenolic content in *G. paniculata* ethanolic extract at 340 nm.

The TPC of the GPEE was 50.4 ± 4.02 mg GAE/100 g d.w. plant material, while the total flavonoid content was 4.83 ± 0.22 mg QE/100 g d.w. plant material. In our study, the HPLC-DAD-ESI MS results identified significant concentrations of hydroxybenzoic acids, hydroxycinnamic acids, and flavones. To identify the polyphenolic compounds in the GPEE, an optimized HPLC/MS method for the identification and quantification of polyphenol compounds was utilized. In total, 11 compounds were identified, of which 9 compounds were flavones, representing approximately 95% of the total phenolics identified via HPLC/MS. The other compounds identified in the extract belonged to the hydroxycinammic acid (2.9%) and hydroxybenzoic acid (2.1%) classes. Among the flavone compounds, luteolin derivatives comprised 51.26%, followed by apigenin derivatives representing 43.77% of the flavones (Figure 1, Table 1).

The GPEE contained one hydroxybenzoic acid derivative (corresponding to peak 1, with *m/z* 139), one caffeic acid derivative (corresponding to peak 2, with *m/z* 344), and nine flavonoid glycosides, the most abundant being luteolin–glucosyl–arabinoside (corresponding to peak 4, with *m/z* 581.287) and apigenin–diglucoside (corresponding to peak 5, with *m*/*z* 595.271); see Table 1.

### 3.2. In Vitro Oxidative Stress Markers

The GPEE exhibited good in vitro antioxidant activity in all conducted tests. The DPPH assay showed a moderate antioxidant activity, the result being between 50 and 100 μg TE/mL, which was superior to TX (*p* = 0.002). The H_2_O_2_ scavenging activity (*p* = 0.001) and FRAP (*p* = 0.001) tests indicated a better performance of the GPEE as compared to TX. The NO scavenging activity of the GPEE was also better when compared to that of quercetin (*p* = 0.01); see Table 2.

### 3.3. In Vivo Oxidative Stress Markers

In comparisons of the oxidative stress parameters between the SZT and CONTROL groups, there were significant increases in oxidant markers, namely, TOS, OSI, MDA, AOPPs, 8-OHdG, NO, 3NT, and AGEs (*p* < 0.001). Meanwhile, the antioxidant defense markers TAC and SH were significantly decreased (*p* < 0.005) (Table 3).

Comparing the effects of the GPEE treatments with the SZT group, we found that TOS and OSI were significantly reduced by GPEE100% and GPEE50% (*p* < 0.001), while GPEE25% had a smaller inhibitory effect on TOS (*p* < 0.05) and OSI (*p* < 0.01). MDA was significantly reduced by GPEE100% and GPEE50% (*p* < 0.001), and a less significant reduction was observed in the GPEE25% group (*p* < 0.01). NO and 3NT were decreased only by GPEE100% (*p* < 0.001) and GPEE50% (*p* < 0.01). The GPEE had no significant effect on 8-OHdG, and AOPPs were lowered only by GPEE100% (*p* < 0.05) and GPEE50% (*p* < 0.01). AGEs were not influenced by the GPEE treatments (*p* > 0.05). The extract improved antioxidant defense through increasing TAC and SH very significantly for GPEE100% (*p* < 0.001) and GPEE25% (*p* < 0.01). Meanwhile, GPEE50% caused a mild increase in SH (*p* < 0.05) and did not influence TAC (*p* > 0.05) (Table 3).

Treatment with metformin significantly reduced TOS, OSI, MDA, 8-OHdG, 3NT, and AGEs. At the same time, metformin increased TAC and SH (*p* < 0.01). When comparing the effects of the GPEE with SZT + M, we found that there were no significant differences (*p* > 0.05) (Table 3).

Treatment with TX reduced TOS, OSI (*p* < 0.05), and MDA (*p* < 0.01) but had no important effects on AOPPs, 8-OHdG, NOx, 3NT, and AGEs (*p* > 0.05) (Table 3). TX, being an antioxidant, increased TAC and SH (*p* < 0.05). Comparing the effects of TX with the GPEE, we found that TX caused more significant reductions in TOS (*p* < 0.05), NOx (*p* < 0.01), and 3NT (*p* < 0.05) than GPEE100%. GPEE50% reduced only NOx and AGE more than TX (*p* < 0.05) (Table 3).

In the GPEE groups, Pearson’s correlation analysis revealed positive correlations with MDA, TOS, and OSI levels (*p* < 0.001); moderate positive correlations with AOPPs, NO, and 3NT (*p* < 0.05); and strong negative correlations with TAC and SH (*p* < 0.01). AOPPs and NO were not correlated with the antioxidant parameters (Table 3).

### 3.4. In Vivo Inflammatory Markers

The inflammatory response was evaluated through measuring NF-κB p65, I1-B, IL-18, and gasdermin D as NLRP3 inflammasome activation markers. When comparing the inflammatory markers in the SZT and CONTROL groups, we found statistically significant increases in NF-κB p65, IL-1β, IL-18, and gasdermin D (*p* < 0.01) in the SZT group. GPEE100% and GPEE50% managed to reduce NF-κB p65 levels moderately (*p* < 0.05), and IL-1β and IL-18 more significantly (*p* < 0.001) (Table 4). 

NF-κB p65 (*p* < 0.05), IL-1β (*p* < 0.001), and IL-18 (*p* < 0.01) were decreased in the metformin group compared to the SZT group. GPEE100% and GPEE50% had a stronger inhibitory activity than metformin only on IL-18 (*p* < 0.01) (Table 4).

When comparing the inflammatory markers in the TX group to the SZT group, we found significant reductions in NF-κB p65 (*p* < 0.05), IL-1β (*p* < 0.001), IL-18 (*p* < 0.001), and gasdermin D (*p* < 0.05). GPEE100% and GPEE50% had a better inhibitory effect on IL-18 than TX (*p* < 0.01) (Table 4).

**Figure 2:**
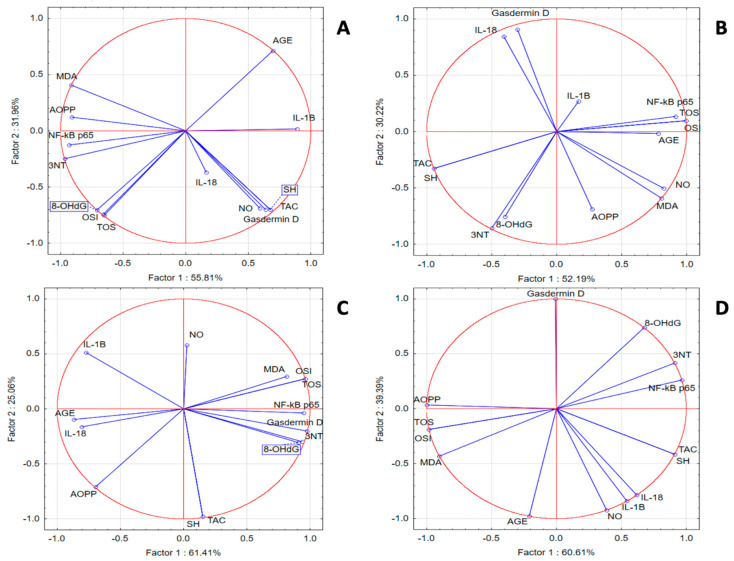
Oxidative stress and inflammatory marker PCA results: (**A**) SZT group; (**B**) GPEE100% group; (**C**) GPEE50% group; and (**D**) GPEE25% group.

In the SZT group, we noticed a statistically significant positive correlation between inflammatory and oxidant markers and a significant negative correlation with TAC and SH. PCA analysis indicated that correlations between IL-18 and IL-1β with OSI, TOS, 8-OHdG, AOPPs, and MDA were present. In the same group, NF-κB p65 was strongly correlated with 3NT and AGE, while gasdermin D was correlated with TAC, SH, and NOx. In the GPEE 100% group, IL-1β and Nκ-kB p65 were correlated with the antioxidant system parameters AOPPs and 8-OHdG; IL-18 was correlated with AGE, TOS, OSI, NOx, and MDA; and gasdermin D was correlated with 3NT. In the GPEE 50% group, IL-1β and IL-18 were correlated with AGE and AOPPs; NF-κB-p65 with NOx; and gasdermin D with TOS, OSI, MDA, 3NT, and 8-OHdG. In the GPEE 25% group, NF-κB p65 was correlated with AGE, 8-OHdG, and 3NT; and IL-1β with NOx (Figure 2).

### 3.5. In Vivo Hypoglycemic, Lipid-Lowering, and Hepatoprotective Effects

SZT induced DM as GLU increased significantly (*p* < 0.001). Animals that did not develop hyperglycemia greater than 250 mg/dl were excluded from the study. GPEE100% and GPEE50% reduced GLU (*p* < 0.05), but the effect was smaller than that of metformin (*p* < 0.01). GPEE25% allowed for an increase in GLU (*p* < 0.01) (Table 5).

Regarding the lipid metabolism profile, TG was reduced by GPEE100% and GPEE50% (*p* < 0.001), whereas TX had a smaller lowering effect (*p* < 0.05). The TyG index, reflecting insulin resistance, increased in SZT animals (*p* < 0.01), but the GPEE, metformin, and TX treatments had no significant effect on it (*p* > 0.05). TC was significantly lowered by GPEE100%, GPEE50%, GPEE25%, and metformin (all *p* < 0.001) (Table 5).

SZT caused liver injury associated with significant increases in AST and ALT levels (*p* < 0.001) and decreased the AST/ALT ratio (*p* < 0.05). Only GPEE100% (*p* < 0.05), GPEE50% (*p* < 0.01), and metformin (*p* < 0.05) reduced AST, while ALT was reduced by GPEE100% (*p* < 0.05), GPEE50% (*p* < 0.01), metformin (*p* < 0.01), and TX (*p* < 0.05). The GPEE, metformin, and TX had no important effect on the AST/ALT ratio when compared to SZT (*p* > 0.05) (Table 5).

SZT-induced DM was associated with a decrease in BW (*p* < 0.01), and only GPEE25% treatment significantly influenced the BW when compared to SZT (*p* < 0.05) (Table 5).

**Figure 3:**
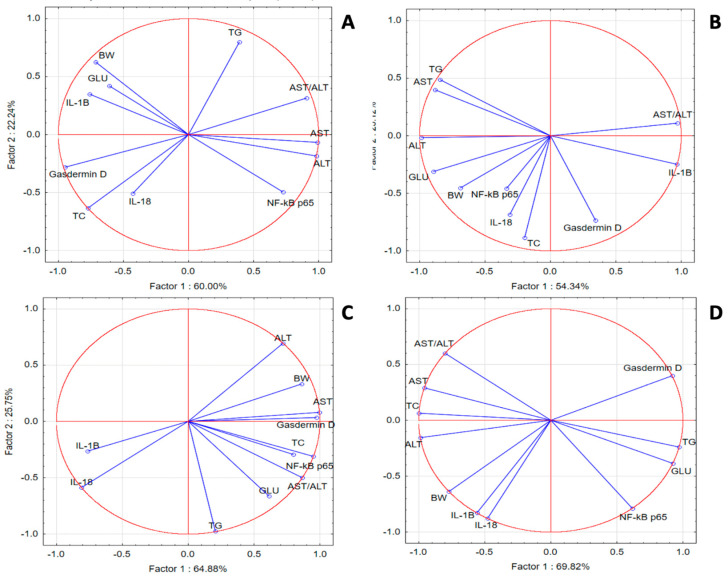
Oxidative stress, metabolic, and liver injury marker PCA results: (**A**) SZT group; (**B**) GPEE100% group; (**C**) GPEE50% group; and (**D**) GPEE25% group.

In the SZT group, PCA revealed weak correlations for IL-18, IL-1 β, gasdermin D, TC and GLU with NF-κB p65, TG, GLU, and transaminases, respectively. In the GPEE100% group, we noticed better correlations for IL-1β with liver injury makers; GLU and TG with NF-κB p65; and liver injury enzymes with TG. In the GPEE50% group, gasdermin D was correlated with liver injury enzymes, GLU, and lipids, while NF-κB p65 was correlated with IL-1β, IL-18, and transaminases. In the GPEE25% group, IL-1β was correlated with transaminases and TC, while NF-κB p65 was correlated with GLU and TG (Figure 3).

## 4. Discussion

The results of the present study revealed that, in an STZ-induced DM model, the GPEE presented important antioxidant and anti-inflammatory activities associated with hypoglycemic, lipid-lowering, and hepato-protective mechanisms. These effects of the GPEE were correlated with the broad spectrum of phenolic compounds that it contains, which may enhance the antioxidant activity of the extract. 

As described above, extensive screening for antidiabetic agents has established natural products as one of the major potential sources of drug discovery [25,26,27,28]. Caryophyllaceae species are rich in phytochemicals and could be an important pharmacological resource for the development of potential antidiabetic agents [29,30,31]. In particular, *Gypsophila* species have been reported to possess antidiabetic, antioxidant, and anti-inflammatory effects [32,33,34].

It is important to identify the phytochemical composition of each plant product, as it could be significantly influenced by the environmental conditions, harvesting time, and extraction method [35]. The yield from plant materials typically varies from year to year, due to the perennial nature of *G. paniculata*. This plant species can be grown in a wide range of soils, from high sand to clay loam and well-drained to friable soil [36]. The vegetable material analyzed in our study was collected during spring from a sea-level region with a mildly acidic soil (pH 6–6.4), a humid continental climate characterized by temperatures ranging from 10–15 °C, and variable precipitation levels in spring. During this season, *G. paniculata* species are typically in their early growth stages. Using this harvesting approach, the variable precipitation levels and lower temperatures may influence the obtained phytochemical composition. 

The TPC of the GPEE, determined through the Folin–Ciocâlteu method, was higher than those determined in other *Gypsophila* species, such as *G. pilulifera*, *G. arrostii*, and *G. simonii* [9,32], while the TFC was similar to those determined in other studies for GPEEs [37]. Phytochemical analysis indicated that the GPEE contained a significant number of phenolic compounds. The most frequently identified compounds were represented by glycosylated flavones, of which the most abundant were luteoline and apigenin derivatives. Previous studies have revealed that luteoline derivatives possess antidiabetic activities through their effects on oxidative stress and proinflammatory status [38]. Apigenin can stimulate insulin secretion, suppress the activity of α-glucosidase, and scavenge ROS [39]. 

For evaluation of the in vitro antioxidant capacity, we used four methods: DPPH, FRAP, NO, and H_2_O_2_ scavenging capacity assays. The GPEE displayed a medium DPPH-reducing power and significant FRAP-reducing, H_2_O_2_, and NO scavenging activity. The in vitro antioxidant capacity of the GPEE was consistent with that reported in other studies [9].

Considering the significant contribution of OS in the pathophysiology of DM and its complications, we chose to evaluate the in vivo effect of the GPEE on serum OS markers in an experimental rat SZT-induced DM model. SZT is a diabetogenic chemical, being a cytotoxic glucose analog that, after administration, accumulates in pancreatic beta cells, causing DNA fragmentation and cell death [40,41,42,43,44]. The main sources of OS in diabetes are represented by several metabolic pathways, including the hexosamine, protein kinase C, glycolytic, and polyol pathways [45,46]. TOS is usually used to estimate the overall oxidation state of the body, while TAC is used to measure the overall antioxidant status. The OSI represents the ratio of TOS to TAC and tends to be a more precise index of OS [45]. The GPEE in all concentrations had a clear impact on TOS and OSI, reducing their values. In addition, GPEE100% and GPEE50% had a significantly better capacity to decrease TOS and OSI when compared to TX and metformin. 

MDA is the dialdehyde of malonic acid and is used as a biomarker of lipid peroxidation, an important process in atherosclerosis and late complications of DM [47,48]. The GPEE managed to significantly reduce the MDA levels at all three concentrations, the results being comparable to groups treated with metformin and TX—our reference antioxidants. 

Chronic hyperglycemia leads to endothelial dysfunction, which involves fatty acid oxidation, impaired nitric oxide (NO) synthesis, OS, inflammation, and altered barrier function [49]. In DM, even short-term exposure to hyperglycemia leads to a selective increase in expression of the iNOS gene, followed by an increase in NO [49,50]. In T2DM, during hyperglycemia and insulin resistance, increased superoxide anion production could also induce overproduction of NO, which, in turn, reacts with superoxide anions and produces peroxynitrite (ONOO^−^), a member of the reactive nitrogen species (RNS) causing nitrosative stress [49,50]. While OS can damage proteins, lipids, and DNA, thus inducing apoptosis, nitrosative stress can cause necrosis [51,52]. GPEE100% and GPEE50% treatments led to a significant decline in NO levels. Moreover, reactive ONOO^−^ can cause protein tyrosine nitration, leading to structural alteration of these proteins and cell disruption. As 3-nitrotyrosine (3NT) is a stable nitrated protein, it can be used as a biomarker for the assessment of oxidative and nitrosative stress in concert with other biomarkers [53]. The GPEE lowered 3NT, with the highest concentration having the best inhibitory activity. 

The most-studied oxidative DNA damage marker is 8-OHdG, which is produced when the endogenous antioxidant network and DNA repair systems are overwhelmed [54]. Elevated 8-OHdG levels indicated a degree of oxidative DNA damage in the SZT group, and the GPEE, metformin, and TX had no significant effects on 8-OHdG.

AOPPs are oxidatively modified protein products, which are the most commonly measured markers of OS; additionally, glycemic control in patients with T1DM or T2DM has been reported to be correlated with AOPP levels [55,56,57,58]. In our study, AOPP concentrations were significantly increased in the SZT group. While GPEE100% and GPEE50% managed to significantly reduce AOPP levels, GPEE25%, TX, and metformin did not lower their concentration.

Hyperglycemia triggers an increased glucose uptake in insulin-independent cells and promotes non-enzymatic glycation of proteins, which can further react to form AGEs. OS is thought to promote the formation of protein–AGEs which, in turn, can act to promote additional OS, forming a vicious circle. AGEs were significantly increased in the SZT group, and only GPEE50% managed to significantly reduce them, with its effect being comparable to that of metformin.

A low antioxidant defense can lead to OS, damaging cells, DNA, and proteins, as well as promoting lipid peroxidation and the development of insulin resistance in the context of DM [59,60,61,62]. TAC and SH were markedly decreased in the SZT group and were significantly increased by the GPEE, similar to TX, indicating that the antioxidant activity of the GPEE is not just related to the reduction of oxidants, but also acts through increased antioxidant levels. 

Chronic inflammation is a prominent characteristic in the natural course of DM, and levels of inflammatory biomarkers are correlated with DM complications [63]. Therefore, we evaluated the anti-inflammatory properties of the GPEE. The transcription factor NF-κB is a critical regulator of immune and inflammatory responses [64]. NF-κB induces the NLRP3 inflammasome and pro-IL-1β expression. Inflammasomes are a group of cytosolic multiprotein complexes produced by immune cells, serving as signaling platforms of the innate immunity associated with tissue damage. Previous studies have shown that the NLRP3 inflammasome plays a vital role in the development of DM and its associated complications [65,66,67,68]. The NLRP3 inflammasome consists of caspase adaptor (ASC), pro-caspase-1, and the NLRP3 protein. Once assembled, the NLRP3 inflammasome activates caspase-1, enabling the processing of pro-IL-1β and pro-IL-18 into their mature bioactive forms, as well as gasdermin D-mediated pyroptosis [65,66,67]. Natural products, including phenols, terpenoids, and alkaloids, have been shown to exhibit significant inhibitory activities against the NLRP3 inflammasome [68,69,70]. STZ increased NF-κB p65, which significantly activated the NLRP3 inflammasome as IL-1β, IL-18, and gasdermin D were increased. Meanwhile, the GPEE exerted anti-inflammatory activity through reducing NF-κB p65, IL-1β, and IL-18, but had a less important effect on gasdermin D. The effect of the GPEE was comparable to that of metformin. Consequently, the antioxidant activity of the GPEE is associated with an anti-inflammatory action.

The GPEE lowered the blood glucose concentration in SZT animals, and treatment with GPEE100% and GPEE50% had a hypoglycemic effect similar to that of metformin.

In patients with DM, high blood TG and TC levels are a consequence of hyperglycemia [71,72]. Lipid-soluble antioxidants such as polyphenols can inhibit cholesterol synthesis and stimulate the expression of LDL receptors [73,74]. GPEE100% and GPEE50% reduced TG and TC levels but did not influence the TyG index, indicating that, at high concentrations, the GPEE has hypolipidemic activity, but cannot influence insulin resistance. The hypolipidemic activity of the GPEE was correlated with its hypoglycemic and ROS reduction effects.

Furthermore, there exists a link between T2DM and metabolic dysfunction-associated fatty liver disease (MAFLD). Glucotoxicity, lipotoxicity, and OS-injured hepatocytes lead to increased liver transaminase release [75,76]. Through reducing GLU, TG, and TC, GPEE100% and GPEE50% caused correlated reductions in AST and ALT, with an effect comparable to that of metformin. 

In the STZ group, the changes in the metabolic parameters GLU, TG, and TC correlated with anthropometric measurements, and BW was found to have decreased. The GPEE, metformin, and TX treatments had no significant influence on the BW, even though they lowered these metabolic markers. 

The limitations of this study included a small sample size, short intervention period, and results based on a single-batch animal model, which may not be accurately extrapolated to human outcomes. Other limitations included the plant material being obtained from a single growing season and the possible associated influences of the environmental conditions. Therefore, the results may not be universally attributed to *G. paniculata* species and future studies are necessary for validation.

## 5. Conclusions

In conclusion, the presented experimental results suggested that the GPEE may serve as an adjuvant pathogenetic therapy in T2DM due to its antioxidant and anti-inflammatory effects. The activities of the GPEE were correlated with its phytochemical composition. The antioxidant and anti-inflammatory activities of the GPEE were associated with reductions in glucose, triglycerides, total cholesterol, and liver injury enzymes—effects that can lower the risk of DM complications. In the STZ-induced DM model, the effects of the GPEE were dose-dependent, with higher doses having a better effect. However, further studies are necessary to validate the presented results.

## Figures and Tables

**Table 1:** The contents of phenolic compounds in *G. paniculata* extract via HPLC (μg/mL).

Peak No.	R_t_ (min)	UV λ_max_ (nm)	[M + H]^+^ (*m*/*z*)	Phenolic Compound	Subclass	Quantity * (μg/mL)
1.	3.23	270	139	2-Hydroxybenzoic acid	Hydroxybenzoic acid	114.600
2.	12.78	332	344	Caffeic acid–glucoside	Hydroxycinnamic acid	152.257
3.	13.81	350.270	727.271	Apigenin–glucosyl–glucosyl–arabinoside	Flavone	725.999
4.	14.04	350.260	581.287	Luteolin–glucosyl–arabinoside	Flavone	1264.852
5.	14.24	340.270	595.271	Apigenin–diglucoside	Flavone	1183.805
6.	15.16	350.260	449.287	Luteolin–glucoside	Flavone	166.615
7.	15.51	350.270	565.271	Apigenin–glucosyl–arabinoside	Flavone	244.841
8.	15.92	340.270	433.271	Apigenin–glucoside	Flavone	140.998
9.	16.25	350.260	463.287	Luteolin–glucuronide	Flavone	590.785
10.	16.65	350.260	611.287	Luteolin–diglucoside	Flavone	545.008
11.	17.25	340.260	773.303	Quercetin–rutinoside–glucoside	Flavone	114.044
				Total phenolics		5243.804

* Quantity expressed as: μg gallic acid equivalent/mL for hydroxybenzoic acids; μg chlorogenic acid equivalent/mL for hydroxycinnamic acids; μg rutin equivalent/mL for flavones.

**Table 2:** In vitro antioxidant test results for *G. paniculata* ethanolic extract.

Sample	DPPH (μgTE/mL)	H_2_O_2_ Scavenging Activity (μgTE/mL)	NO Scavenging Activity (μgQE/mL)	FRAP (mgTE/mL)
*G. paniculata* IC50	94.31 ± 10.5	102.07 ± 20.03	76.33 ± 6.77	98.06 ± 17.9
Trolox IC50	11.2 ± 1.7	24.23 ± 3.12	-	12.07 ± 2.04
Quercetin IC50	-	-	20.58 ± 3.67	-
*p*-value	0.002	0.001	0.003	0.001

DPPH, α,α-diphenyl-β-picrylhydrazyl; NO, Nitric oxide; H_2_O_2_, Hydrogen peroxide; FRAP, Ferric-reducing antioxidant power; QE, Quercetin equivalent; TE, Trolox equivalent.

**Table 3:** Effects of *G. paniculata* on oxidative stress markers in rat streptozotocin-induced diabetes mellitus model.

Parameters	Control	SZT	GPEE100%	GPEE50%	GPEE25%	SZT + M	SZT + TX
TOS (μM/L)	18.05 ± 3.74	42.12 ± 6.23 ^aaa^	14.02 ± 3.46 ^bbb,cc,d^	19.13 ± 3.04 ^bbb^	21.31 ± 2.70 ^b^	25.02 ± 4.25 ^bbb^	26.11 ± 1.95 ^b^
TAC (mM/L)	1.09 ± 0.00	1.084 ± 0.0014 ^a^	1.087 ± 0.0011 ^bbb,ccc,ddd^	1.085 ± 0.0003 ^ccc,ddd^	1.086 ± 0.0011 ^bb,ccc,ddd^	1.09 ± 0.002 ^bb^	1.09 ± 0.00 ^b^
OSI	16.57 ± 3.43	38.83 ± 5.72 ^aaa^	12.87 ±1.19 ^bbb,cc^	17.57 ± 3.31 ^bbb^	19.58 ± 4.93 ^bb^	22.91 ± 3.87 ^bbb^	23.99 ± 1.99 ^b^
MDA (nM/L)	2.28 ± 0.05	3.26 ± 0.24 ^aaa^	2.24 ± 0.21 ^bbb^	2.14 ± 0.26 ^bbb^	2.39 ± 0.17 ^bb^	2.14 ± 0.17 ^bbb^	2.25 ± 0.20 ^bbb^
AOPPs (μM/L)	126.93 ± 10.70	169.27 ± 15.59 ^aaa^	124.78 ± 29.86 ^b^	135.72 ± 14.35 ^bb^	166.48 ± 22.64	161.70 ± 26.19	165.69 ± 38.15
8-OHdG (ng/mL)	44.81 ± 3.3	48.87 ± 3.02	45.32 ± 2.13	50.24 ± 7.34	53.52 ± 2.39	44.49 ± 5.34 ^b^	42.56 ± 3.49
NOx (μM/L)	30.39 ± 5.71	60.88 ± 1.35 ^aaa^	44.29 ± 4.56 ^bbb,c,dd^	44.20 ± 9.64 ^bb,c,d^	59.51 ± 4.44	57.73 ± 7.96	58.48 ± 6.94
3NT (ng/mL)	42.68 ± 7.09	62.74 ± 9.70 ^a^	34.26 ± 2.48 ^bb,c,d^	48.04 ± 6.49 ^b^	45.66 ± 6.74 ^b^	44.39 ± 5.68 ^b^	64.44 ± 4.95
AGEs (ng/mL)	11.49 ± 1.40	13.21 ± 0.38 ^a^	12.68 ± 0.65	11.45 ± 1.14 ^b,d^	13.39 ± 1.85	10.85 ± 1.70 ^b^	14.20 ± 1.27
SH (μM/L)	324.67 ± 22.21	259.67 ± 21.50 ^a^	417.80 ± 36.19 ^bb^	373.29 ± 11.12 ^b^	443.00 ± 43.03 ^bb^	507.8 ± 149.08 ^bb^	426.00 ± 40.88 ^b^

Values are expressed as mean ± SD (standard deviation). ^a^ vs. CONTROL: ^a^ *p* < 0.05, ^aaa^ *p* < 0.001; ^b^ vs. SZT: ^b^ *p* < 0.05, ^bb^ *p* < 0.01, ^bbb^ *p* < 0.001; ^c^ vs. SZT + M: ^c^ *p* < 0.05, ^cc^ *p* < 0.01, ^ccc^ *p* < 0.001; ^d^ vs. SZT + TX: ^d^ *p* < 0.05, ^dd^ *p* < 0.01, ^ddd^ *p* < 0.001; SZT, Streptozotocin; SZT + TX, Streptozotocin + Trolox; SZT + M, Streptozotocin + Metformin; GPEE, *G. paniculata* ethanol extract; TOS, Total oxidative status; TAC, Total antioxidant capacity; OSI, Oxidative stress index; MDA, Malonyldialdehide; AOPPs, Advanced oxidation protein products; 8-OHdG, 8-hydroxydeoxyguanosine; NOx, Nitrites and nitrates; 3NT, 3-nitrotyrosine; AGEs, Advanced glycation end-products; SH, Total thiols.

**Table 4:** Effects of *G. paniculata* ethanol extract on inflammatory markers in rat streptozotocin-induced diabetes mellitus model.

Parameters	Control	SZT	GPEE100%	GPEE50%	GPEE25%	SZT + M	SZT + TX
NF-κB p65 (pg/mL)	165.11 ± 33.25	306.33 ± 59.84 ^a^	252.18 ± 43.10 ^b^	240.96 ± 33.18 ^b^	253.23 ± 49.34	230.54 ± 18.45 ^b^	222.58 ± 46.43 ^b^
IL-1β (pg/mL)	23.75 ± 5.67	132.5 ± 35.05 ^a^	20.62 ± 5.82 ^bbb^	26.00 ± 2.63 ^bbb^	23.12 ± 1.78 ^bbb^	19.58 ± 3.99 ^bbb^	27.91 ± 3.15 ^bbb^
IL-18 (pg/mL)	16.30 ± 2.93	59.45 ± 6.16 ^aa^	0.01 ± 0.00 ^bbb,ccc,ddd^	4.51 ± 1.74 ^bbb,cc,dd^	5.02 ± 1.32 ^bbb,cc,dd^	23.08 ± 2.75 ^bb^	11.74 ± 2.25 ^bbb^
Gasdermin D (ng/mL)	5.87 ± 1.05	9.50 ± 1.65 ^aa^	8.22 ± 1.27	7.74 ± 2.41	8.01 ± 2.30	9.38 ± 1.10	6.52 ± 1.35 ^b^

Values are expressed as mean ± SD (standard deviation). ^a^ vs. CONTROL: ^a^ *p* < 0.05, ^aa^ *p* < 0.01; ^b^ vs. SZT: ^b^ *p* < 0.05, ^bb^ *p* < 0.01, ^bbb^ *p* < 0.001; ^c^ vs. SZT + M: ^c^ *p* < 0.05, ^cc^ *p* < 0.01, ^ccc^ *p* < 0.001; ^d^ vs. SZT + TE: ^d^ *p* < 0.05, ^dd^ *p* < 0.01, ^ddd^ *p* < 0.001; SZT, Streptozotocin; SZT + TX, Streptozotocin + Trolox; SZT + M, Streptozotocin + Metformin; GPEE, *G. paniculata* ethanol extract; NF-κB p65, Nuclear factor kappa B p65 subunit; IL-1β, Interleukin-1 beta; IL-18, Interleukin-18.

**Table 5:** Effects of *G. paniculata* ethanol extract on metabolic, liver injury and anthropometric markers in rat streptozotocin-induced diabetes mellitus.

Parameters	Control	SZT	GPEE100%	GPEE50%	GPEE25%	SZT + M	SZT + TX
GLU (mg/dL)	102.17 ± 9.91	432.67 ± 36.14 ^aaa^	382.80 ± 28.73 ^b^	374.71 ± 83.05 ^b^	529.00 ± 13.75 ^bb,cc^	371.60 ± 58.93 ^bb^	412.5 ± 39.36
TG (mg/dL)	62.53 ± 12.86	128.91 ± 8.24 ^aaa^	82.83 ± 2.39 ^bb,d^	87.00 ± 12.92 ^bb,d^	136.02 ± 17.50	132.04 ± 4.36	116.62 ± 7.53 ^b^
TyG index	4.38 ± 0.12	5.46 ± 0.11 ^aa^	5.18 ± 0.28	5.19 ± 0.3	5.59 ± 0.15	5.40 ± 0.06	5.39 ± 0.11
TC (mg/dL)	70.24 ± 5.44	123.63 ± 6.66 ^aaa^	101.83 ± 3.91 ^bb^	89.44 ± 3.40 ^bbb,d^	100.29 ± 1.41 ^bb^	95.25 ± 8.49 ^bbb^	112.36 ± 12.75
BW (g) change	13.33 ± 1.45	62.16 ± 4.22 ^aa^	74.80 ± 8.49	64.00 ± 5.37	67.00 ± 22.6 ^b^	59.0 ± 5.19	59.25 ± 7.8
AST (U/L)	69.01 ± 3.46	157.98 ± 23.86 ^aaa^	115.12 ± 35.76 ^b^	106.99 ± 26.90 ^bb^	190.70 ± 24.04	128.13 ± 38.14 ^b^	137.79 ± 42.05 ^a^
ALT (U/L)	63.33 ± 8.49	161.57 ± 27.91 ^aaa^	116.63 ± 20.03 ^b^	97.58 ± 3.73 ^bb,c^	160.52 ± 38.32 ^c^	128.51 ±20.5 ^bb^	131.07 ± 28.54 ^a,b^
AST:ALT	1.11 ± 0.10	0.98 ± 0.47 ^a^	0.97 ± 0.17	1.10 ± 0.14	1.21 ± 0.17	0.98 ± 0.10	0.97 ± 0.09

Values are expressed as mean ± SD (standard deviation). ^a^ vs. CONTROL: ^a^ *p* < 0.05, ^aa^ *p* < 0.01, ^aaa^ *p* < 0.001; ^b^ vs. SZT: ^b^ *p* < 0.05, ^bb^ *p* < 0.01, ^bbb^ *p* < 0.001; ^c^ vs. SZT + M: ^c^ *p* < 0.05, ^cc^ *p* < 0.01; ^d^ vs. SZT + TX: ^d^ *p* < 0.05; SZT, Streptozotocin; SZT + TX, Streptozotocin + Trolox; SZT + M, Streptozotocin + Metformin; GPEE, *G. paniculata* ethanol extract; AST, Aspartate aminotransferase; ALT, Alanine aminotransferase; AST:ALT ratio, Aspartate to alanine aminotransferase ratio; TC, Total cholesterol; TG, Triglyceride; GLU, Glucose; TyG index, Triglyceride to glucose index; BW, Body weight.

## Data Availability

Data are available only for reviewers until the first author defends her Ph.D. thesis.
